# Asthma hospitalisation trends from 2010 to 2015: variation among rural and metropolitan Australians

**DOI:** 10.1186/s12889-017-4704-y

**Published:** 2017-09-18

**Authors:** Daniel Terry, Shalley Robins, Samantha Gardiner, Ruby Wyett, Md Rafiqul Islam

**Affiliations:** 1 Department of Rural Health, The University of Melbourne, 49 Graham Street, Shepparton, VIC 3630 Australia; 2 Melbourne Medical School, The University of Melbourne, Level 2 West, Medical Building (181), Melbourne, VIC 3010 Australia; 3 Ballarat Base Hospital, 1 Drummond St N, Ballarat Central, VIC 3350 Australia; 4 Goulburn Valley, Health, Graham St, Shepparton, VIC 3630 Australia; 5 School of Health and Social Development, Deakin University, Burwood Highway, Burwood, VIC 3125 Australia

**Keywords:** Asthma, Hospitals, Metropolitan, Rural, General practice, Self-management, Chronic illness

## Abstract

**Background:**

Asthma remains a leading cause of illness, where primary care can assist to reduce hospitalisations through prevention, controlling acute episodes, and overall management of asthma. In Victoria, Asthma hospitalisations were as high as 3.1 hospitalisations per 1000 population in 1993–94. The primary aims of this study are to: determine if changes in asthma hospitalisations have occurred between 2010 and 2015; determine the key factors that impact asthma hospitalisation over time; and verify whether rural and urban asthma hospitalisations are disparate. A secondary aim of the study is to compare 2010–2015 results with asthma data prior to 2010.

**Methods:**

Hospital separation data from 1 July 2010 to 30 June 2015 were obtained through the Victorian Admitted Episodes Dataset and other agencies. Data included sex, age, Local Government Area, private or public patient, length of stay, and type of discharge. Asthma and predictor variables were analysed according to hospital separation rates after adjusting for smoking and sex. Hierarchical multiple regression examined the association between asthma and predictor variables.

**Results:**

During the study period, 49,529 asthma hospital separations occurred, of which 77.5% were in metropolitan hospitals, 55.4% hospital separations were aged 0–14 years, and 21.7% were privately funded. State-wide hospital separations were 1.85 per 1000 population and were consistently higher in metropolitan compared to rural areas (1.93 vs 1.64 per 1000 population). When data among metropolitan adults aged 15 and over were analysed, an increase in the proportion of smokers in the population was reflected by an increase in the number of hospital separations (Adj OR 1.035). Further, among rural and metropolitan children aged 0–14 the only predictor of asthma hospital separations was sex, where metropolitan male children had higher odds of separation than metropolitan females of the same age (Adj OR 4.297). There was no statistically meaningful difference for separation rates between males and females in rural areas.

**Conclusions:**

We demonstrated a higher overall hospital separation rate in metropolitan Victoria. For children in metropolitan areas, males were hospitalised at higher rates than females, while the inverse was demonstrated for children residing in rural areas. Therefore, optimising asthma management requires consideration of the patient’s age, gender and residential context. Primary health care may play a leading role in increasing health literacy for patients in order to improve self-management and health-seeking behaviour.

## Background

Asthma is characterised by chronic airway inflammation that is associated with reversible airway narrowing and obstruction due to a response to diverse and unrelated stimuli [1, 2]. As a chronic airway condition, asthma remains a significant disease internationally with Australia having the highest prevalence of any other country where 10.2% or 2.3 million people were diagnosed with the ambulatory care-sensitive condition (ACSC) in 2011–2012 [3–5]. Asthma hospitalisation rates are one of the ten National Indicators for population monitoring of Asthma in Australia, particularly as many continue to seek asthma care at hospital. Despite this, hospitalisation rates for asthma in Australia are relatively low compared to other countries [1, 6].

Seeking hospital care may be due to a number of reasons including limited access to primary care; primary health care costs; avoidance coping mechanisms; dislike of, poor compliance to, or improper use of asthma medications; lack of a written asthma action plan; or poor attitudes towards self-management [1, 7–9]. It has been suggested that admission rates for ACSCs, such as asthma may be a key measure for primary health care quality where ambulatory care can aid in reducing the risk of hospitalisation through illness prevention, controlling acute episodes, and overall management of the chronic condition [10]. However, due to the characteristics and variability of asthma, including the diverse triggers for severe exacerbations (including viral infections and extreme allergen exposure, such as during thunderstorm epidemics), hospitalisations may sometimes be appropriate [11].

One Australian study highlighted the number of hospitalisations for asthma were as high as 53,907 (2.86 hospital separations per 1000 population) in 1998–99, dropping to 36,703 (1.69 hospital separations per 1000 population) in 2008–09 [1]. Hospital separations are defined as “the administrative process, by which, a hospital records the cessation of treatment and/or care and/or accommodation of a patient” and this is how the utilisation of hospital services are commonly measured [12]. At the state level, hospital separations rates for asthma in Victoria were 3.1 hospital separations per 1000 population in 1993–94 and dropped to 1.96 hospital separations per 1000 population in 2001–02 [13, 14]. Both national and state data indicates that there may be improvements in the affordability, accessibility, and capacity of primary health care to manage asthma through preventative measures such as medications and written asthma care planning [1].

Despite developments in primary care, medication quality and self-management, the prevalence of asthma continues to be affected by environmental, physical and social factors. These factors include socioeconomic status, sex, smoking, Aboriginality, and the degree of remoteness of residency [1]. For example, living and working in rural areas has the potential for greater exposure to fumes, dust, pesticides, and herbicides which may contribute to chronic respiratory illnesses such as asthma [15–19]. Conversely, these potential risk factors may in fact be protective among rural populations, where environmental conditions such as these result in the development of a protective response in the individual, reducing their likelihood of developing asthma [15–19]. However, living in metropolitan areas may contribute to the development of asthma through exposure to particulate matter and air pollution from traffic emissions, as well as indoor biological irritants such as animal dander and dust mites [19].

Regardless of the many origins or contributing factors of asthma, it remains a complex disease with devastating outcomes that requires greater engagement with primary health care, conscientious self-management practices, and appropriate medication compliance [8]. The current evidence and literature remains insightful and informative regarding the improvement in asthma hospitalisation rates while guiding primary and tertiary care policy and protocols. As such, the primary aims of this study were to: examine the most recently available hospital separation data to determine if changes in asthma hospitalisations had occurred between 2010 and 2015; determine what key factors impacted asthma hospitalisation over this time; and verify whether rural and urban asthma hospitalisations were disparate. A secondary aim of the study was to also compare 2010–2015 findings with asthma data prior to 2010.

## Methods

The state of Victoria is marginally smaller than the United Kingdom in size with more than 5.35 million people living across the 237,269 km^2^ area. It encompasses 79 local government areas (LGA) consisting of 1 Borough, 39 Shires, 7 Rural Cities, and 32 City Councils that range from 20 to 22,213 km^2^ in size. Further, Victorian population densities across the local government areas range from 0.47 to 4413.26 people per square kilometre, and within the state, there are over 300 public and private hospitals including district hospitals and bush nursing services [20].

Hospital separation data for a five-year period from 1 July 2010 to 30 June 2015 were obtained from the Victorian Admitted Episodes Dataset (VAED) that collects data from all hospital separations in the State. Data included sex, age (5-year age groups), LGA or region of residence, private or public patient, length of stay (LOS) in bed days, type of discharge, and diagnosis on admission according to the ICD-10-AM. Asthma as the principal diagnosis was identified using the ICD-10-AM codes J45.0, J45.1, J45.8, J45.9, and J46.0. Ethical approval for the research was obtained in March 2015.

Others sources of data included the 2011–2015 Census of Population and Housing Victorian and Regional Health Profile 2012 data, which contains amalgamated data from Australian Bureau of Statistics, Commonwealth Department of Health and Ageing, Department of Health, Department of Planning and Community Development, VicHealth, and the Medical Directory of Australia [20]. This dataset provided data on each LGA in Victoria and included 2011–2015 population (to accommodate for demographic evolution of the population over the five-year period), Aboriginal or Torres Strait Islanders population, average income, unemployment rate, percentage of current smokers, and rate of general practitioners (GPs) per 1000 population.

In addition, data were compared to previous asthma data and studies that examined asthma in Victoria [1, 11, 13]. Further, additional data regarding each Victorian LGA in 2011–2015 was sourced from the Australian Bureau of Statistics and further separated into deciles and quintiles for analysis [21]. This data included the Index of Relative Socio-economic Disadvantage (IRSD) which is used to measure the socio-economic disadvantage according to geographic areas; the Socio-Economic Indexes for Areas (SEIFA) that ranks areas in Australia according to relative socio-economic advantage and disadvantage; the Index of Economic Resources (IER) which examines the financial aspects of socio-economic advantage and disadvantage; and the Index of Education and Occupation (IEO) which reflects the educational and occupational level of various communities. Lastly, the Accessibility and Remoteness Index of Australia 2011 (ARIA+) and the Australian Statistical Geography Standard (ASGS) categories were sourced to score the level of geographical of remoteness. ARIA+ score is calculated using road distance from goods and services based on population size, while ASGS is a more comprehensive measure [22]. In Victoria each LGA was scored using the ARIA+ as highly accessible (0–0.2), accessible (0.2–2.4), and moderately accessible (2.4–5.92) [23].

### Data analysis

Population figures at the LGA level were used to calculate hospital separation rates across the 79 LGAs in Victoria. Hospital separations were age- and sex-standardised using corresponding yearly population estimates for each LGA. Age groups were also examined separately for children (0–14 years of age) and adults (≥15 years of age), given the marked differences in exacerbation between these two groups and a greater proportion of hospital expenditure occurring among the 0–14 years of age group [6, 11].

Asthma and predictor variables were analysed according to hospital separation rates by LGA using Statistical Package for the Social Sciences (SPSS, Version 22.0). Hierarchical multiple regression was used to examine the association between asthma separations and several of predictor variables including percentage of smokers, sex, GPs per 1000 population, percentage of Aboriginal and Torres Strait Islanders, SEIFA, IRSD, IER, IEO, ASGS and ARIA+ score. In addition, multivariable weighted least squares regression was also performed to account for any violations of assumption of equal variance of observations between LGA populations after adjusting for percentage of smokers, sex. Significance was determined at two-tailed *p* ≤ 0.001.

## Results

During the study period, there were 49,529 total hospital separations for Asthma in Victoria between the financial years 2010–11 and 2014–15, of which 38,369 (77.5%) occurred at metropolitan hospitals, 27,534 (55.4%) were aged 0–14 years of age, and 11,045 (21.7%) were privately funded hospital separations. Females represented 9423 (34.2%) of hospital separations for children 0–14 years of age, while females made up 15,019 (68.4%) of all hospital separations among adults aged 15–85+ years of age (Table 1).

**Table 1:** Descriptive data associated with asthma hospital separations from 2010 to 11 to 2014–15

Factor	Number	Percentage	Incidence rate (persons per 1000 population)	95% CI	Number	Percentage	Incidence rate (persons per 1000 population)	95% CI
	0–14 year olds				15–85 + year olds			
Females	9423	34.2	3.82	3.76–3.86	15,019	68.4	1.30	1.29–1.31
Metro residence	21,780	79.1	5.81	5.76–5.85	13,096	75.4	1.14	1.13–1.15
Privately insured patients	4999	18.2	0.98	0.98–0.99	5350	24.3	0.23	0.23–0.24
Emergency admissions	27,136	98.6	5.34	5.34–5.35	18,086	82.3	0.79	0.79–0.80
ARIA+(quintiles)								
0 (≤ 0.1)	6264	19.3	6.39	6.303–6.49	3875	20.5	0.83	0.81–0.85
1 (0.1–0.6)	5353	18.9	5.57	5.48–5.66	4113	20.6	0.87	0.86–0.89
2 (0.7–1.3)	6226	20.2	6.02	5.93–6.11	4877	18.8	1.14	1.12–1.16
3 (1.4–2.0)	4162	20.1	4.08	4.00–4.15	4937	20.0	1.09	1.07–1.11
4 (2.1–5.0)	3654	21.3	3.37	3.30–3.43	4208	19.9	0.93	0.91–0.95
Population density (quintiles**)**								
0 (≤ 2.77)	4044	21.4	3.72	3.71–7.34	4676	19.9	1.03	1.03–1.04
1 (2.78–8.15)	6994	20.6	6.67	6.66–6.69	4539	20.7	0.96	0.96–0.97
2 (8.16–126.57)	6300	19.7	6.29	6.27–6.31	5032	18.9	1.17	1.17–1.18
3 (126.58–1480.79)	6384	19.1	6.55	6.54–6.57	4167	19.4	0.94	0.94–0.95
4 (≥1480.80)	5308	19.0	5.49	5.47–5.50	3617	20.7	0.77	0.76–0.77
IRSD (quintiles)								
0 (≥962.90)	5783	18.2	6.24	6.14–6.34	5093	21.3	1.05	1.05–1.06
1 (962.91–981.20)	5638	19.8	5.58	5.49–5.67	4908	20.2	1.06	1.06–1.07
2 (981.21–1001.80)	5565	19.9	5.49	5.40–5.58	4603	20.8	0.97	0.97–0.98
3 (1001.81–1055.20)	5395	18.2	5.31	5.22–5.39	4258	19.0	0.98	0.98–0.99
4 (≤1055.21)	5147	21.8	4.64	4.56–4.72	3148	18.5	0.75	0.75–0.76
IEO (quintiles)								
0 (≥943)	6021	18.8	6.30	6.20–6.39	4123	20.0	0.91	0.90–0.91
1 (944–964)	5655	20.4	5.44	5.35–5.53	4670	20.1	1.02	1.00–1.04
2 (965–989)	5847	21.1	5.44	5.36–5.53	4559	21.1	0.95	0.95–0.96
3 (990–1049)	5421	19.8	5.38	5.29–5.46	4801	20.5	1.03	1.03–1.04
4 (≤1050)	4584	19.6	4.59	4.51–4.67	3857	18.2	0.93	0.93–0.94
IER (quintiles)								
0 (≥963)	6194	20.1	6.04	5.95–6.13	438	18.0	1.09	1.09–1.10
1 (964–975)	5267	20.3	5.10	5.02–5.18	5422	20.4	1.16	1.16–1.17
2 (976–999)	4975	19.6	4.99	4.90–5.07	4586	20.1	1.00	1.00–1.01
3 (1000–1022)	6112	20.0	6.09	6.00–6.18	4172	21.5	0.85	0.85–0.86
4 (≤1023)	4922	19.8	4.89	4.81–4.98	3392	19.8	0.75	0.75–0.76
GP/1000 population (quintiles)								
0 (≤8.77)	6498	19.7	6.49	6.40–6.59	4119	17.5	1.05	1.03–1.07
1 (8.78–10.00)	5245	19.9	5.17	5.08–5.25	5169	20.8	0.84	0.70–0.98
2 (10.10–10.90)	5585	20.2	5.44	5.35–5.53	4370	20.9	0.92	0.90–0.94
3 (10.91–12.40)	5116	19.9	5.06	4.98–5.15	4479	19.9	0.68	0.65–0.71
4 (≥12.41)	5084	20.1	4.96	4.88–5.04	3873	20.9	0.79	0.75–0.83

Hospitalisation rates were higher among the male population aged 0–14 years with males 0–4 years of age having an incidence rate of 13.76 per 1000 population, while incidence rates increased among the female population aged 15–85 + years as outlined in Table 2.

**Table 2:** Descriptive data associated with asthma hospital separation rates from 2010 to 11 to 2014–15

Age (years)	n	Percentage	Incidence rate (per 1000 population)	95% CI	n	Percentage	Incidence rate (per 1000 population)	95% CI
	Female				Male			
0–4	5888	32.1%	6.88	6.86–6.90	12,441	67.9%	13.76	13.72–13.80
5–9	2560	36.9%	3.16	3.15–3.17	4369	63.1%	5.13	3.45–6.80
10–14	975	42.9%	1.21	1.21–1.22	1298	57.1%	1.53	0.49–2.57
15–19	888	60.0%	1.03	1.02–1.03	592	40.0%	0.65	−0.30-1.61
20–24	1064	66.1%	1.06	1.05–1.06	545	33.9%	0.52	−0.45-1.49
25–29	996	68.5%	0.95	0.95–0.96	457	31.5%	0.43	−0.49-1.34
30–34	1153	69.2%	1.18	1.17–1.18	512	30.8%	0.52	−0.50-1.55
35–39	1314	70.2%	1.31	1.31–1.32	557	29.8%	0.57	−0.51-1.65
40–44	1241	69.7%	1.22	1.22–1.22	539	30.3%	0.55	−0.49-1.59
45–49	1188	66.2%	1.23	1.23–1.24	606	33.8%	0.65	−0.40-1.70
50–54	1019	60.7%	1.10	1.10–1.11	660	39.3%	0.74	−0.25-1.73
55–59	1033	68.7%	1.25	1.24–1.25	471	31.3%	0.59	−0.46-1.64
60–64	1061	65.2%	1.40	1.39–1.40	567	34.8%	0.78	−0.34-1.89
65–69	991	74.0%	1.67	1.66–1.68	349	26.0%	0.61	−0.61-1.83
70–74	832	73.4%	1.78	1.78–1.79	302	26.6%	0.68	−0.58-1.94
75–79	790	71.0%	2.04	2.03–2.05	322	29.0%	0.98	−0.37-2.33
80–84	728	74.1%	2.20	2.19–2.21	255	25.9%	1.04	−0.37-2.44
85+	721	79.5%	2.11	2.10–2.12	186	20.5%	1.04	−0.33-2.41

**Fig. 1:**
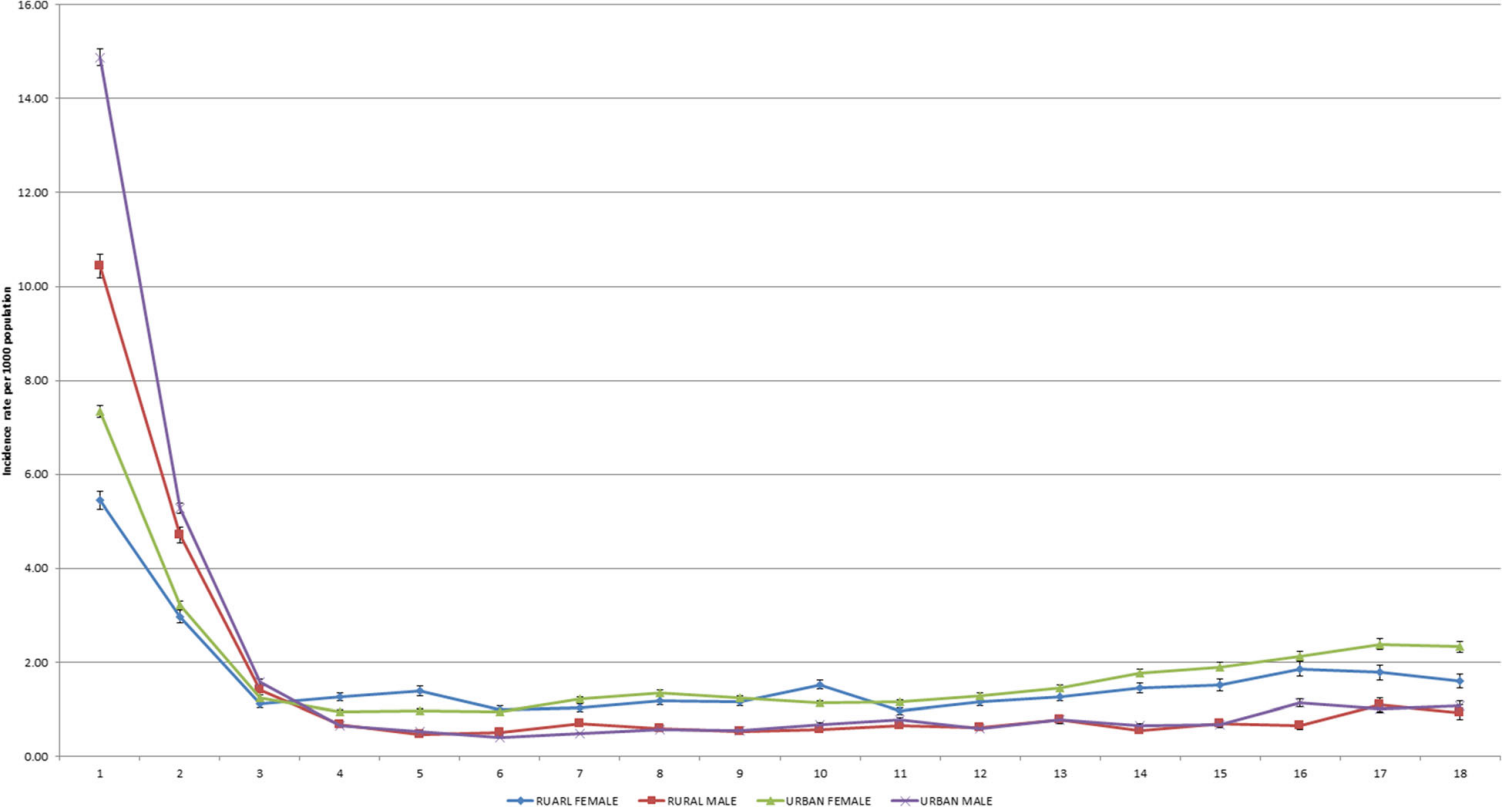
Hospital separation rate per 1000 population according to 5 year age group separated by sex and region of residence across 79 Local Government Areas (LGA) in Victoria. To obtain standardised separations rates per 1000 population, hospital separations were divided by the corresponding population estimates for each year according to gender, age group and LGAs to obtain standardised separations rates per 1000 population. Data shows highest incident rate of hospital separations occurred among metropolitan males aged 0–4 years followed by rural males aged 0-4 years, while rural and metropolitan females had higher incident rates among those aged 15–85+ years

Further, when examining incidence rates between metropolitan and rural areas and age groups, it was noted that metropolitan males 0–4 years of age had the highest incidence overall (Fig. 1).

The average state-wide LOS during the same time period was 1.92 bed days (95% CI: 1.90–1.94). The average LOS significantly differed between rural and metropolitan areas at 2.17 (95% CI: 2.13–2.17) and 1.85 bed days (95% CI: 1.82–1.87) respectively, *p* = 0.001. The lowest state-wide average LOS occurred in 2014–15 with 1.80 bed days (95% CI: 1.76–1.85) with rural and metropolitan average LOS 2.03 (95% CI: 1.93–2.14) and 1.75 bed days (95% CI: 1.70–1.79) respectively, *p* = 0.001.

However, what was noted was that the overall state average bed days decreased from 1.92 to 1.80 days between 2010 and 2015. Using the 2014–15 data it represents a 33.5% decrease in length of stay since 1999–2000 [13]. However, compared to this previous data, it was found that hospital admission rates for asthma differed between rural and metropolitan areas. For example, between 1999 and 2000 and our 2010–2015 findings, rural admission rates decreased much more than the admission rates observed in metropolitan areas. Specifically, hospital admission rates in rural areas decreased 41.5% between 1999 and 2000 and 2014–15, while hospital admission rates increased 5.6% in metropolitan areas [13].

Among all Victorian children aged 0–14 years, the average LOS was 1.30 bed days (95% CI: 1.28–1.32), while the number of bed days was significantly higher in rural areas than in metropolitan areas at 1.48 (95% CI: 1.45–1.52) and 1.25 bed days (95% CI: 1.23–1.28), *p* = 0.001. Similar findings were observed among adults aged 15–85+ years with a state-wide average LOS of 2.72 bed days (95% CI: 2.66–2.77) while rural populations stayed in hospital for longer than their metropolitan counterparts, staying for an average of 2.95 (95% CI: 2.86–3.04) and 2.64 bed days (95% CI: 2.58–2.70) respectively, *p* = 0.001.

Victorian hospital separations rates for asthma over the five-year period was 1.85 (95% CI: 1.84–1.86) per 1000 population. However, when examining the difference between children and adults, it was found that among children 0–14 year of age, the state-wide hospital separations rate was 5.43 (95% CI: 5.38–5.46) per 1000 population, and in adults aged 15–85+, it was 0.97 (95% CI: 0.96–0.98) per 1000 population.

Hospital separation rates were significantly higher in metropolitan areas compared with rural areas, with rates being 1.93 (95% CI: 1.92–1.94) and 1.64 (95% CI: 1.62–1.66) per 1000 population, respectively, *p* = 0.001. Similarly, the hospital separation rate in metropolitan children aged 0–14 years was 5.81 (95% CI: 5.76–5.85) per 1000 population which was significantly higher than that of rural children of the same age who were hospitalised at a rate of 4.34 per 1000 population (95% CI: 4.26–4.40), *p* = 0.001. Conversely, among adults aged 15–85+ years, hospital separations rates per 1000 population were not significantly different between metropolitan and rural areas (*p* = 0.83).

**Fig. 2:**
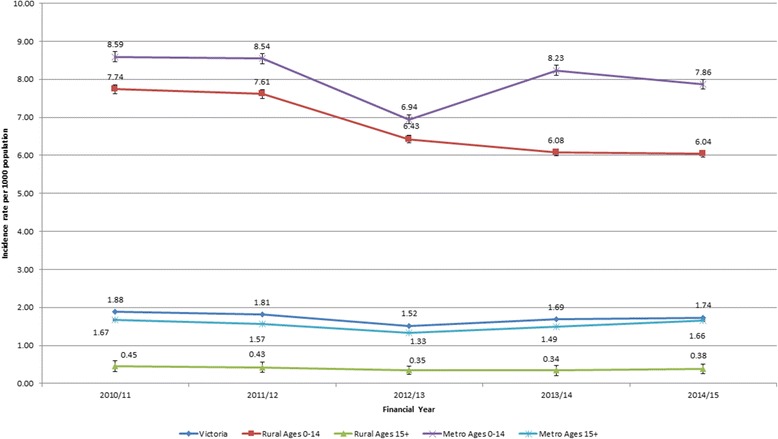
Hospital separation rate per 1000 population according to year of hospital separation, region of residence, and age differences. To obtain standardised separations rates per 1000 population for each year, hospital separations were divided by the corresponding population estimates for each year according region of residence and according to hospital separations among children (0–14 years of age) and adults (≥15 years). This is due to the marked differences in asthma exacerbation between these two groups. Data shows state-wide hospital separations rates for asthma between 2010 and 11 and 2014–15 remained relatively stable from 1.88 to 1.74 hospital separations per 1000 population between 2010 and 11 and 2014–15. While rural and metropolitan admissions among patients aged 15 years and older remained relatively unchanged over the same time. Data also shows among metropolitan groups aged 0–14 years there was a significant decrease from 8.59 to 7.86 hospital separations per 1000 population from 2010 to 11 to 2014–15, *p* = 0.001. While there was a decrease from 7.74 to 6.04 per 1000 population was observed in rural areas, *p* = 0.001. Overall, the hospital separation rates for asthma were consistently higher in metropolitan compared to rural Victoria over the five-year period, *p* = 0.001

Figure 2 shows the variation in standardised hospital separations rates for asthma between 2010 and 11 and 2014–15 with the overall Victorian rate remaining relatively stable from 1.88 (95% CI: 1.87–1.88) hospital separations per 1000 population in 2010–11 to 1.74 (95% CI: 1.73–1.74) hospital separations per 1000 population in 2014–15. This represents a 10.2% decrease in the average hospital admission since 1999–2000 [1, 11, 13].

Specifically, this data highlights in Table 3 that admissions among metropolitan patients aged 15 years and older remained relatively similar between 2010 and 2015. However, among metropolitan patients aged 0–14 years old, there was a significant decrease from 8.59 (95% CI: 8.58–8.61) to 7.86 (95% CI: 7.85–7.88) hospital separations per 1000 population from 2010 to 11 to 2014–15, *p* = 0.001.

**Table 3:** Regression analysis associated with asthma and rurality from 2010 to 11 to 2014–15

Predictors	OR(adj)	95% CI	*p*-value	OR(adj)	95% CI	*p*-value
	Aged 0–14			Aged 15–85+		
	Metropolitan			Metropolitan		
Smokers (percentage)	0.947	0.831–1.080	.405	1.035	1.016–1.052	.001*
Sex (male)	4.297	2.054–8.998	.001*	1.045	1.151–1.054	.360
GP ratio (per 1000)	0.720	0.092–5.618	.742	0.808	0.567–1.153	.224
ABTSI	0.159	0.003–9.079	.353	0.640	0.318–1.287	.197
Private insurance	0.002	0.000–1.848	.072	1.035	0.315–3.397	.953
SEIFA	2.328	0.569–9.516	.224	1.057	0.829–1.347	.642
IRSD	0.710	0.283–1.786	.447	1.026	0.875–1.203	.738
IER	0.641	0.405–1.014	.057	0.977	0.903–1.058	.549
IEO	0.731	0.336–1.590	.410	0.887	0.776–1.014	.077
ARIA+	0.926	0.076–11.314	.950	0.955	0.620–1.473	.827
ASGC	0.523	0.086–3.161	.460	0.940	0.689–1.283	.683
Population density Km^2^	1.000	0.999–1.001	.896	1.000	1.000–1.000	.954
	Rural			Rural		
Smokers (percentage)	1.023	0.911–1.080	.691	1.006	0.974–1.039	.700
Sex (male)	0.446	0.277–1.395	.001*	1.068	0.938–1.217	.313
GP ratio (per 1000)	1.326	0.322–5.618	.688	1.234	0.807–1.885	.321
ABTSI (percentage)	1.275	0.698–9.079	.418	1.025	0.856–1.228	.780
Private insurance	0.613	0.004–1.848	.844	2.046	0.454–9.207	.341
SEIFA	0.567	0.146–9.516	.401	1.042	0.694–1.564	.840
IRSD	0.931	0.231–1.786	.918	1.012	0.667–1.536	.953
IER	1.697	0.784–2.014	.173	0.915	0.726–1.153	.441
IEO	1.146	0.706–1.590	.572	1.020	0.882–1.179	.785
ARIA+	1.502	0.523–11.314	.439	1.303	0.950–1.788	.097
ASGC	0.276	0.039–3.161	.190	0.551	0.307–1.010	.046
Population density Km^2^	1.014	1.001–1.027	.008	1.000	0.997–1.003	.998

*p ≤ 0.001

On the other hand, the decrease in the rates of hospital separations from 7.74 (95% CI: 7.72–7.76) to 6.04 (95% CI: 6.02–6.060) per 1000 population was observed in rural areas, *p* = 0.001. Overall, the hospital separation rates for asthma were consistently higher in metropolitan areas compared to rural Victoria over the five-year period, *p* = 0.001.

In addition, when examining hospital separations between ARIA+ regions of highly accessible, accessible, and moderately accessible for ages 0–14 the only significant difference was between highly accessible and moderately accessible areas where rates varied from 6.06 (95% CI: 5.21–6.90) to 3.29 (95% CI: 2.67–3.92) hospital separations per 1000 population, respectively (*p* < 0.001). However, when examining hospital separations between ARIA+ regions of highly accessible, accessible, and moderately accessible for ages 15–85+ there was no significant difference between hospital separations per 1000 population.

Multiple regression analyses showed no significant predictors of asthma hospital separation rates at the LGA level and when data were analysed according to metropolitan or rural LGAs, again there were no significant predictors of asthma hospital separation rates adjusting for smoking and sex. The variations in asthma hospital separation rates between rural and urban Victoria were further examined among children (0–14 years) and adults (15–85 + years).

In the 0–14 years of age, sex was the only significant predictor of hospital separations in both rural and metropolitan areas with Adj OR 4.3 (95% CI 2.05–8.99) and Adj OR 0.44 (95% CI 0.28–1.39) respectively. There were further differences between rural and metropolitan areas, where the percentage of smokers was found to be a significant predictor for asthma hospitalisations among 15–85+ year olds in metropolitan LGAs Adj OR 1.03 (95% CI 1.02–1.05), but not in rural LGAs Adj OR 1.02 (95% CI 0.97–1.04).

## Discussion

We demonstrated an overall stable rate in hospital separations for the data reviewing period with a higher incidence in metropolitan areas compared to rural areas. Male children 0–4 years of age had the highest hospital separation rates while females of all age groups from 15 years or over were hospitalised at consistently higher rates than their male counterparts. Our study demonstrated that Victorian asthma hospital separations decreased between 2010 and 2015 and represented a 10.2% decrease in the average hospital admission since 1999–2000 [13]. This decline is in line with the marked reduction of Australia wide asthma hospitalisations between 1998 and 9 and 2002–3, especially amongst children, which had plateaued between 2002 and 03 and 2008–09 [1, 11, 13]. Further, the overall average LOS decreased between 2010 and 2015 and represented a 33.5% decrease in LOS over the same 15 year period [13]. However, it was found that overall hospital separation rates of asthma in rural and metropolitan areas differed to previous data, where rural separation rates decreased much more than the separation rates observed in metropolitan areas [13].

This may suggest there has been a shift among rural and regional residents regarding health seeking behaviours and the management of asthma [10]. Difficulties faced by rural populations in accessing hospital or urgent care services may have prompted rural Victorians to better engage with local primary health care providers in order to improve asthma self-management [24, 25]. Conversely, rural residents may simply not seek care at the hospital level when required, but rather use other coping strategies, such as self-managing asthma episodes as they occur [26]. Further, all-cause hospitalisations may have also contributed to pressure on asthma hospitalisations and even length of stay among those admitted with asthma; however, the reduction of asthma separations may be more due to effective long-term or preventative asthma management that occurs among primary care providers [1]. This may, in part, explain the difference observed between metropolitan and rural asthma admissions rates.

One specific change of note was the announcement, in the 2008–09 Australian Federal budget regarding the design and implementation of a new four-year program that would be built into the current Asthma Management Plan. This was to be achieved by the Department of Health in collaboration with the National Asthma Council Australia and the Asthma Foundations of Australia [27]. In the 2010–11 budget that followed it was indicated the aim of the redesign was to achieve proactive management of respiratory conditions and best practice treatment while developing the skills of the primary health care workforce through respiratory education programs [28]. Further, a major revision of Australian asthma guidelines was launched in 2014 [11], followed by an extensive implementation program by NPS MedicineWise in primary care that focussed on key messages from the new guidelines; both of these may have impacted on asthma hospitalisations in the final year of the period we examined. In addition, by the time the guidelines were launched, there was already evidence of a major increase in dispensing of inhaled corticosteroid-containing medications over the previous 10 years [29].

These factors may provide some insight into the lower rates of hospital admissions being observed, particularly in rural areas after 2012–13, and may in part explain the marked decrease in metropolitan and rural hospital admissions among 0–14 year olds in 2012–13. Despite this, there may be other factors, such as meteorological phenomena including weather and environmental stimuli [30, 31]. Further, back-to-school factors, particularly after long vacations, are known to be associated with epidemics of asthma hospitalisations among school-aged children and can fluctuate from year-to-year [32]. These factors may potentially explain in the reduction of hospital admissions for asthma year-to-year, specifically observed among 0–14 year olds in 2012–13.

The challenge is that these explanations do not provide insight into the higher admission rates observed among those aged 0–14 years of age in metropolitan areas. It is evident that higher hospitalisation rates may occur in metropolitan communities as they have experienced greater levels of direct (including maternal exposure) to outdoor traffic air pollution leading to poor immune competence among children [33]. Further, it is suggested that there is greater exposure to indoor ambient air pollution among metropolitan residents than their rural counterparts since 1999–2000. However, there is limited evidence to suggest this level of exposure has changed over time [13, 34]. When examining those aged 15–85+ years and older in metropolitan areas, the only predictor variable associated with increased rates of hospital admission was the percentage smokers [33]. Across metropolitan Victoria it was demonstrated that the higher the percentage of smokers the higher the odds (Adj OR 1.03) of hospital admissions for asthma would occur in those aged 15 years and older, while among rural residents aged 15 years and older there were no predictors identified.

When examining metropolitan and rural data dichotomously among the 0–14 year age group, it was further highlighted that being male was a significant predictor of hospital admissions among metropolitan residents. These findings supported the evidence that being male in a metropolitan area lead to higher odds (Adj OR 4.3) of hospital admissions for asthma than their female counterparts as metropolitan males are more susceptible to asthma [35]. This analysis took into account the higher prevalence of asthma in males than females, but it was found that rural males within the same age group had lower odds (Adj OR 0.45) of hospital separations than females which is statistically insignificant.

In rural settings, other than sex, there were no significant predictors of asthma hospital admissions among those aged 0–14 years and no predictors among adults aged 15 and above. Among the data, there was a high level of variability in terms of the predictors that impact hospital admissions in rural areas. This may be caused by other unexamined factors such as cultural diversity, obesity, diet, atopic syndrome, levels of self-efficacy due to service quality, or the various asthma phenotypes that are triggered by multiple genetic and environmental factors [25, 33, 36]. It is these diverse factors that may occur differently according to the various lifestyles and exposure patterns that can take place in diverse rural settings [19]. This heterogeneity between rural communities may contribute toward the diversity of asthma triggers and subsequent hospitalisations that were observed.

In contrast to popular assumptions and current understanding [33], this study has highlighted that socioeconomic status, the level of education and occupation were not predictive of rates of asthma hospitalisations, neither in rural nor in metropolitan areas. These findings, in conjunction with other findings, provide insight into the direction of future research; in particular research should aim to examine rural exposure, metrological factors, genetic contributors, patient beliefs and self-efficacy, and the impact of primary health care on asthma management at the rural community level with keeping female gender in consideration.

Limitations of the study include using retrospective rather than prospective asthma hospitalisations data, while only using acute episodic data regarding this chronic disease. Rates of atopy as a secondary or tertiary diagnosis were scant or not present which limited the examination of atopic syndrome as an indicator of asthma. Further, hospital readmission data or specific postcode data for asthma hospital separations were not available for each admitted patient episode. Therefore the rates of patient readmission and analysis of data at smaller and more specific locales could not be performed in this instance.

Furthermore, the study period ending in mid-2015 limited the capacity to examine the impact of the new asthma guidelines and National Prescribing Service program on rates of asthma hospitalisation among communities and across the state. However, the data used was the most recent data available and remains a full dataset of asthma hospital separations across the whole state of Victoria, providing insight into variations in asthma hospitalisations across the 79 LGAs. The data may not be transferable to all populations, such as very remote populations; however, the results of the study do provide insights into asthma and the underlying differences between metropolitan and rural contexts.

## Conclusion and policy implications

The study suggests that the current national asthma management plan may have made some inroads in the reduction in asthma hospitalisation rates, particularly in rural and regional areas and among children aged 0–14 years of age. Further, the study may indicate the implications of nation-wide interventions that seek to improve the management of asthma through primary health care providers, carers and patients. This is particularly among children 0–14 year olds in rural areas where these factors have had a direct impact on hospital seeking among those experiencing an asthmatic exacerbation. Thus, focused public health efforts and health services interventions concerning asthma management must improve the quantity and quality of primary health care delivery in order to lead to greater self-efficacy.

Overall, the aim of such an approach would be to encourage greater health literacy among Victorian parents and school-aged children concerning asthma recognition and management by enabling individuals, both young and old, to seek assistance at the level of primary care. This may in turn lead to improved self-management of their condition, as seen globally [8, 37]. Additional goals include improving the quality of life among community members while further reducing the demands placed on hospital services. Although health care access barriers remain diverse and complex, future research is required to specifically identify the unique factors that both impact and reduce rural asthma hospitalisations.

## Abbreviations

ARIA +: Accessibility and Remoteness Index of Australia 2011
ASGS: Australian Statistical Geography Standard
GP: General practitioner
ICD-10-AM: International Statistical Classification of Diseases and Related Health Problems, Tenth Revision, Australian Modification
IEO: Index of Education and Occupation
IER: Index of Economic Resources
IRSD: Index of Relative Socio-economic Disadvantage
LGA: Local Government Area
SEIFA: Socio-Economic Indexes for Areas
VAED: Victorian Admitted Episodes Dataset
